# Supplementary material for: Diet Quality and Resilience through Adulthood: A Cross-Sectional Analysis of the WELL for Life Study
Source: Nutrients. 2024 May 31;16(11):1724. doi: 10.3390/nu16111724 (PMC11174593; doi:10.3390/nu16111724)
Supplement: Supplementary file 1 [file nutrients-16-01724-s001.zip › nutrients-3020179-supplementary.pdf]

Supplemental Information:

Bivariate Figures:

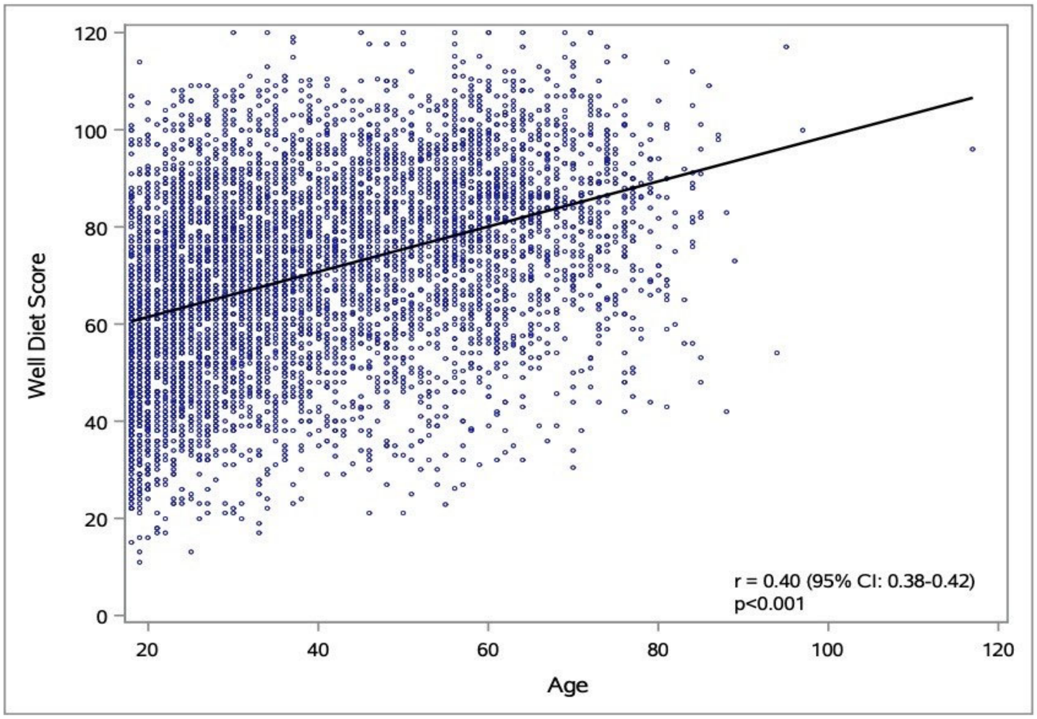

Figure S1a. Age vs WELL Diet Score (scatterplot).

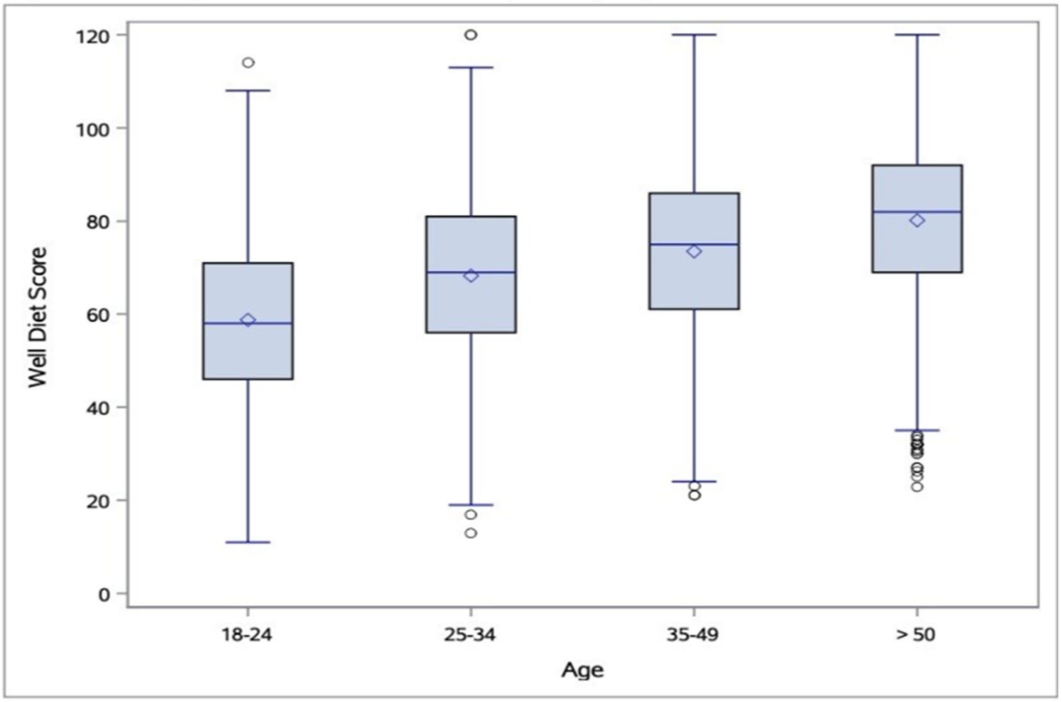

Figure S1b. Age vs WELL Diet Score (box and whiskers).

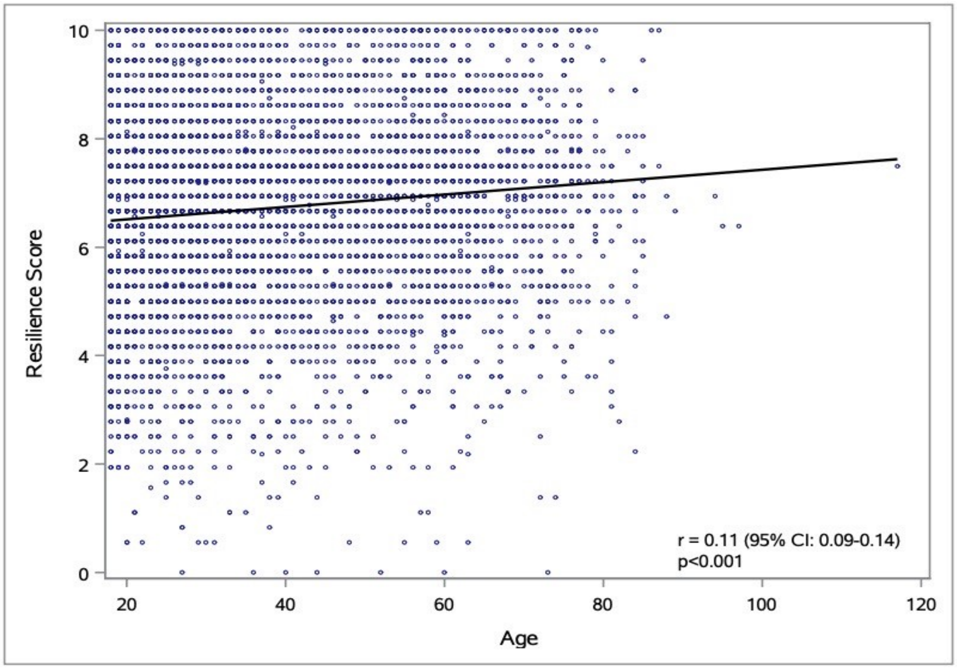

Figure S2a. Age vs. Resilience Score (scatterplot).

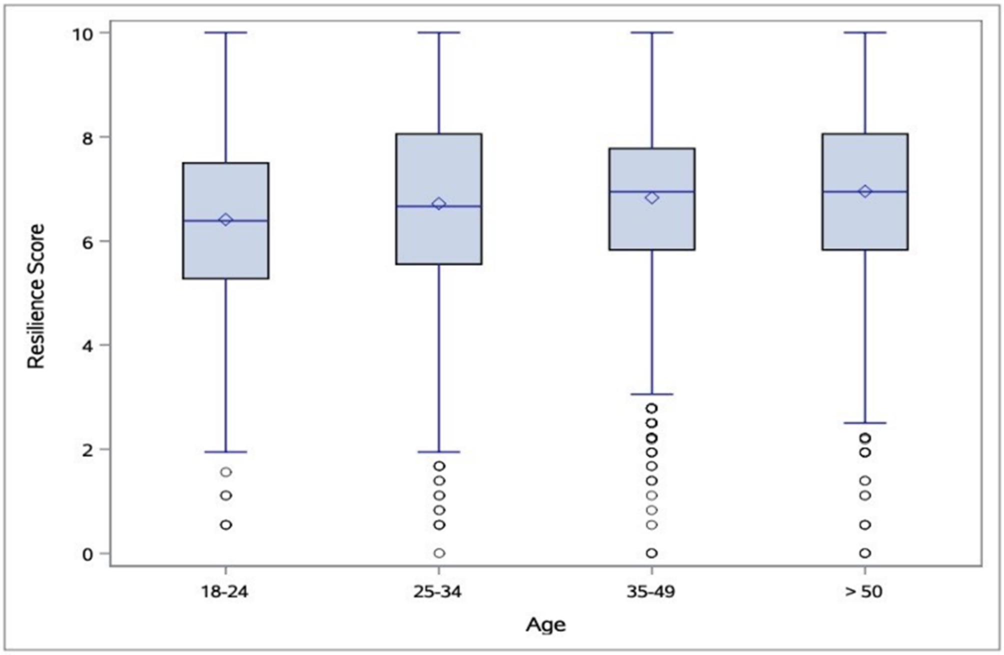

Figure S2b. Age vs. Resilience Score (box and whiskers).

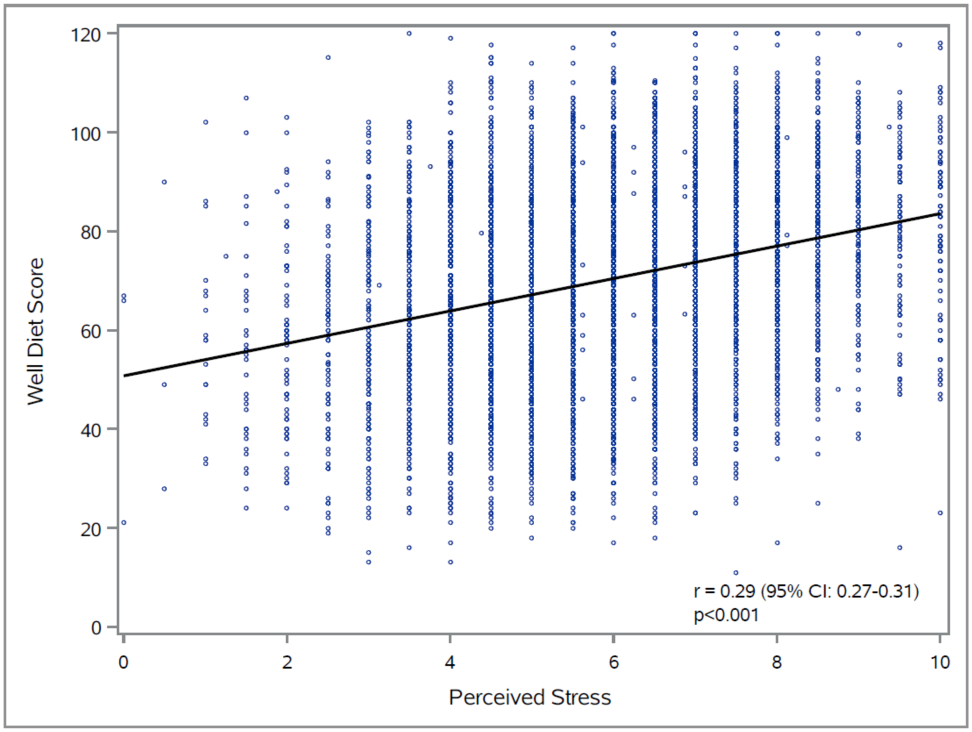

Figure S3a. Perceived Stress vs. WELL Diet Score (scatter plot).
Note* Perceived Stress in reverse scored (meaning higher WELL Diet Score lower stress)

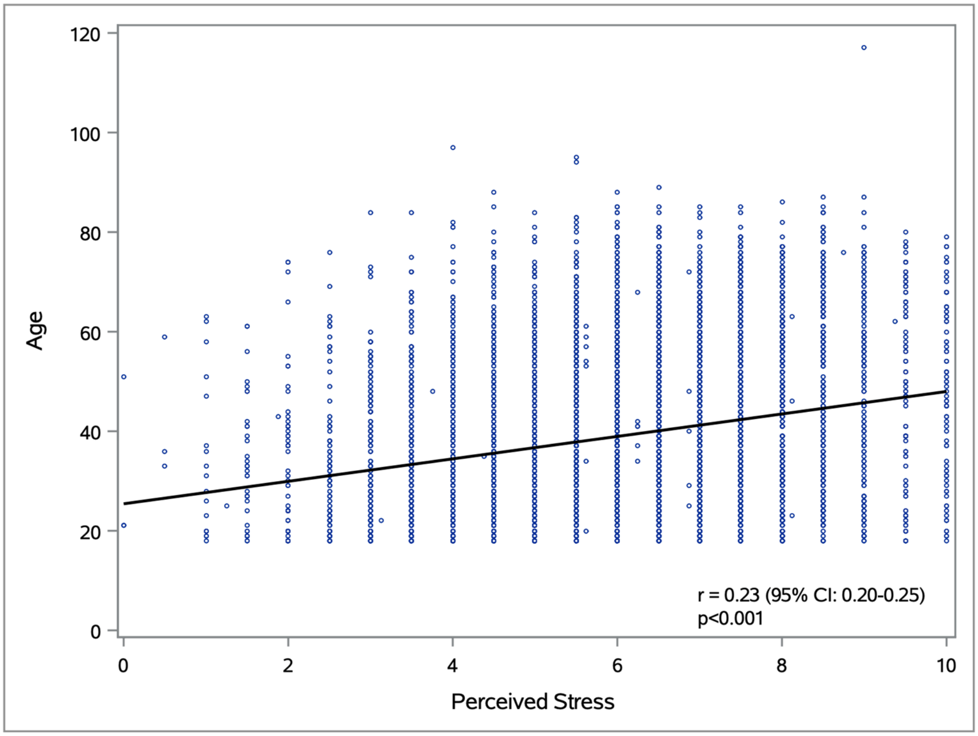

Figure S3b. Perceived Stress vs. Age (scatter plot**).**
Note* Perceived Stress in reverse scored (meaning older participants reported lower stress)

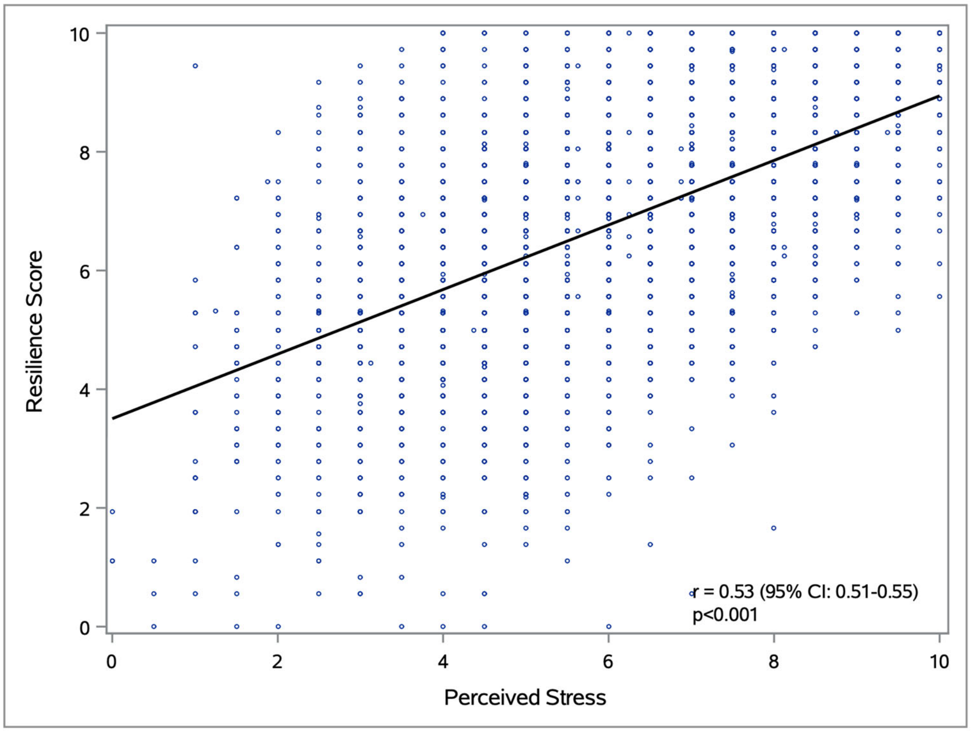

Figure S3c. Perceived Stress vs. Resilience Score (scatter plot).
Note* Perceived Stress in reverse scored (meaning greater resilience lower stress)

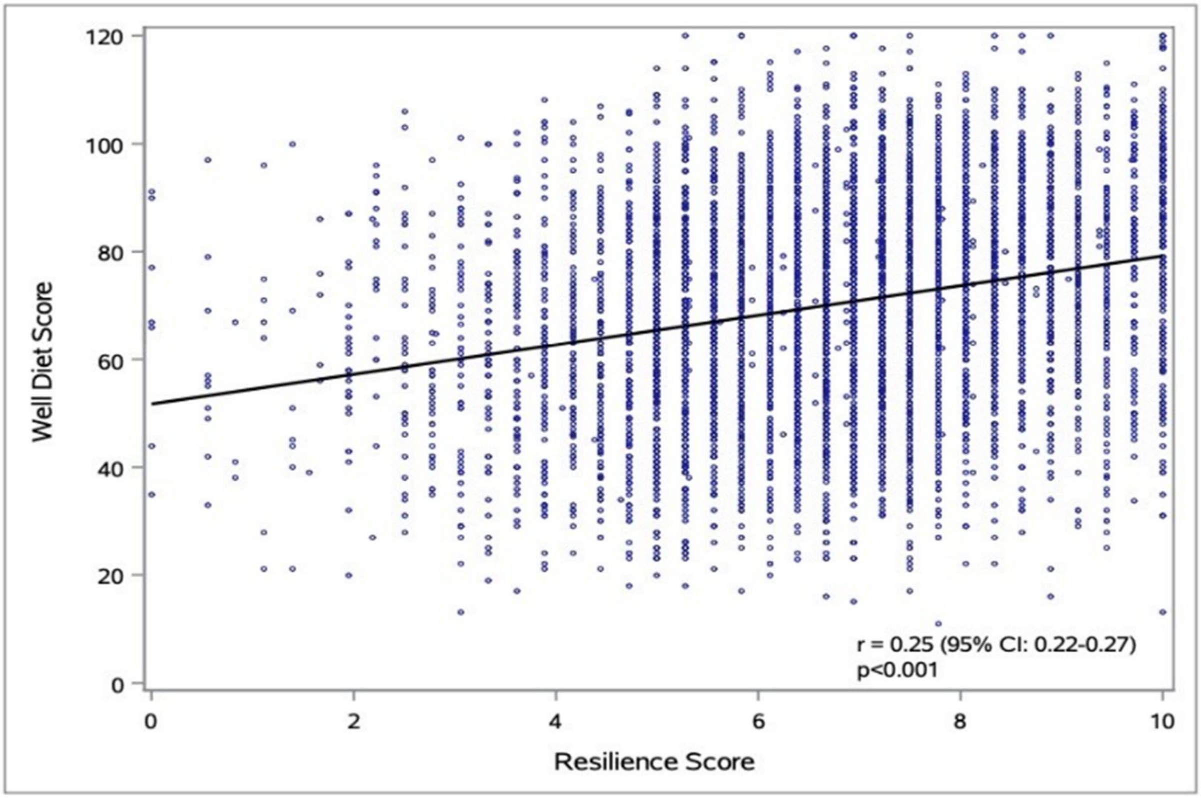

Figure S4. WELL Diet Score vs. Resilience Score (scatterplot).

Appendix SA: WELL Resilience Items adapted from the Brief Resilience Scale (BRS) and the Connor Davidson Resilience Scale (CD-RISC)

[Adapted from BRS] How confident are you that you can bounce back quickly after hard times? 5, Extremely confident | 4, Very confident | 3, Moderately confident | 2, Slightly confident | 1, Not at all confident

> [Original question from BRS] Item 1. I tend to bounce back quickly after hard times. Strongly Agree | disagree | neutral | agree | strongly agree

1. [Adapted from CD-RISC] How confident are you that you can adapt to change? 5, Extremely confident | 4, Very confident | 3, Moderately confident | 2, Slightly confident | 1, Not at all confident

   [Original question from CD-RISC] Item V1. I am able to adapt to change. Rated from "not true at all" to "true nearly all the time".

2. [Adapted from CD-RISC] How confident are you that you can deal with whatever comes your way? 5, Extremely confident | 4, Very confident | 3, Moderately confident | 2, Slightly confident | 1, Not at all confident

   [Original question from CD-RISC] Item V4. I can deal with whatever comes my way. Rated from "not true at all" to "true nearly all the time".

3. [Adapted from CD-RISC] How confident are you that you can see the humorous side of problems? 5, Extremely confident | 4, Very confident | 3, Moderately confident | 2, Slightly confident | 1, Not at all confident

[Original question from CD-RISC] Item V6. I see the humorous side of things. Rated from "not true at all" to "true nearly all the time".

[WELL] How confident are you that you can overcome obstacles? 5, Extremely confident | 4, Very confident | 3, Moderately confident | 2, Slightly confident | 1, Not at all confident

[Adapted from CD-RISC] How confident are you that you can stay focused under pressure? 5, Extremely confident | 4, Very confident | 3, Moderately confident | 2, Slightly confident | 1, Not at all confident

[Original question from CD-RISC] Item V14. Under pressure, I can focus and think clearly. Rated from "not true at all" to "true nearly all the time".

[Adapted from CD-RISC] How confident are you that you can think of yourself as a strong and resilient person? 5, Extremely confident | 4, Very confident | 3, Moderately confident | 2, Slightly confident | 1, Not at all confident

[Original question from CD-RISC] Item V17. I think of myself as a strong person. Rated from "not true at all" to "true nearly all the time".

[Adapted from CD-RISC] How confident are you that you can manage any unpleasant feelings that you might have? 5, Extremely confident | 4, Very confident | 3, Moderately confident | 2, Slightly confident | 1, Not at all confident

[Original question from CD-RISC] Item V19. I can handle unpleasant feelings. Rated from "not true at all" to "true nearly all the time".

[Adapted from CD-RISC] How confident are you that you can not get disheartened by setbacks? 5, Extremely confident | 4, Very confident | 3, Moderately confident | 2, Slightly confident | 1, Not at all confident

[Original question from CD-RISC] Item V16. I am not easily discouraged by failure. Rated from "not true at all" to "true nearly all the time".
